# What a Pandemic Has Taught Us About the Potential for Innovation in Rural Health: Commencing an Ethnography in Canada, the United States, Sweden, and Australia

**DOI:** 10.3389/fpubh.2021.768624

**Published:** 2021-12-07

**Authors:** Samuel Petrie, Dean Carson, Paul Peters, Anna-Karin Hurtig, Michele LeBlanc, Holly Simpson, Jaymie Barnabe, Mikayla Young, Mara Ostafichuk, Heidi Hodge, Justin Gladman, Matilda Smale, Manueal Gonzalez Garcia

**Affiliations:** ^1^Spatial Determinants of Health Laboratory, Department of Health Sciences, Carleton University, Ottawa, ON, Canada; ^2^Department of Epidemiology and Global Health, Umeå University, Umeå, Sweden; ^3^School of Business and Law, Central Queensland University, Rockhampton, QLD, Australia; ^4^College of Medicine and Public Health, Flinders University, Adelaide, SA, Australia

**Keywords:** innovation, rural, health systems, virtual care, COVID-19

## Abstract

The COVID-19 pandemic coincided with a multi-national federally funded research project examining the potential for health and care services in small rural areas to identify and implement innovations in service delivery. The project has a strong focus on electronic health (eHealth) but covers other areas of innovation as well. The project has been designed as an ethnography to prelude a realist evaluation, asking the question *under what conditions can local health and care services take responsibility for designing and implementing new service models that meet local needs?* The project had already engaged with several health care practitioners and research students based in Canada, Sweden, Australia, and the United States. Our attention is particularly on rural communities with fewer than 5,000 residents and which are relatively isolated from larger service centres. Between March and September 2020, the project team undertook ethnographic and auto-ethnographic research in their own communities to investigate what the service model responses to the pandemic were, and the extent to which local service managers were able to customize their responses to suit the needs of their communities. An initial program theory drawn from the extant literature suggested that “successful” response to the pandemic would depend on a level of local autonomy, “absorptive capacity,^*^” strong service-community connections, an “anti-fragile^†^” approach to implementing change, and a realistic recognition of the historical barriers to implementing eHealth and other innovations in these types of rural communities. The field research in 2020 has refined the theory by focusing even more attention on absorptive capacity and community connections, and by suggesting that some level of ignorance of the barriers to innovation may be beneficial. The research also emphasized the role and power of external actors to the community which had not been well-explored in the literature. This paper will summarize both what the field research revealed about the capacity to respond well to the COVID-19 challenge and highlight the gaps in innovative strategies at a managerial level required for rapid response to system stress.

^*^Absorptive Capacity is defined as the ability of an organization (community, clinic, hospital) to adapt to change. Organizations with flexible capacity can incorporate change in a productive fashion, while those with rigid capacity take longer to adapt, and may do so inappropriately.

^†^Antifragility is defined as an entities' ability to gain stability through stress. Biological examples include building muscle through consistent use, and bones becoming stronger through subtle stress. Antifragility has been used as a guiding principle in programme implementation in the past.

## Introduction

The aim of this research was to describe how health and care services in small rural areas in Australia, Sweden, Canada, and the United States of America (USA) engaged with their communities in the early part (March-October 2020) of the COVID-19 pandemic. Throughout the field research, two broad frameworks were developed—one focusing on *what* health and care services (and other actors) were doing, and one on *how* services were able to respond well to the challenges the pandemic presented. This paper focuses on “success stories,” hoping to provide positive inspiration for communities of this type. While the focus on success could obscure the full perspective of how rural health systems responded to the COVID-19 pandemic, the motivation to focus on these successes is to disseminate knowledge about what works where and for whom in a sparsely researched area. There were, of course, also examples of responses that the research team perceived to be poor or insufficient, and assessing these are part of our future directions.

**Figure 1:**
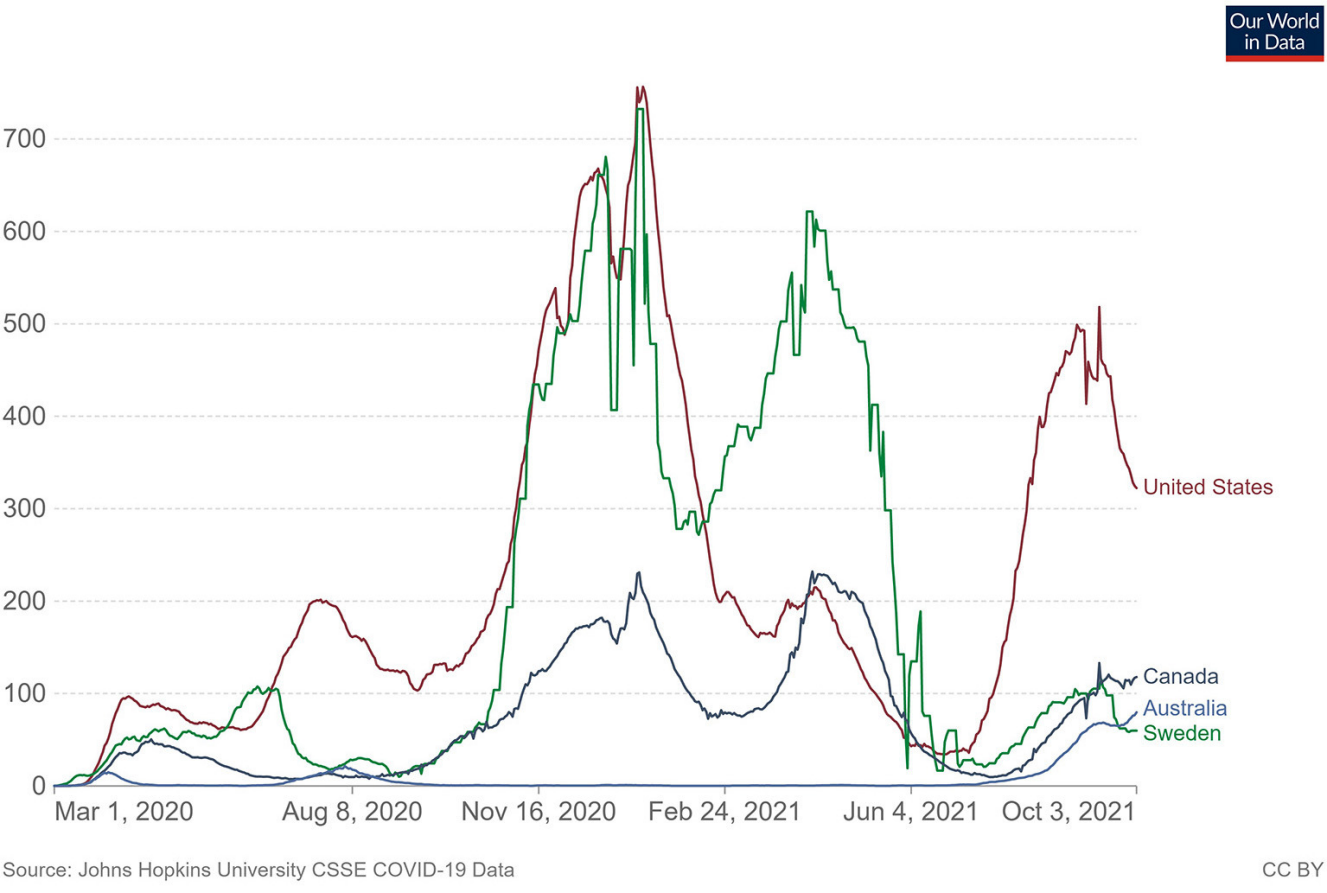
Daily new confirmed COVID-19 cases per million people Shown is the rolling 7-day average. The number of confirmed cases is lower than the number of actual cases; the main reason for that is limited testing. Source: Johns Hopkins University CSSE COVID-19 data.

The geographical context for the research was central to its undertaking. Our research interest has long been in understanding how health services operate in small rural settings, where service sustainability is challenged by relatively small population sizes (the largest towns within a functional service area having fewer than 5,000–7,000 inhabitants) and intermediate distances to larger service centres (1, 2). By intermediate distances we mean that larger centres are accessible by road without necessitating (although they often do involve) overnight stays, but not daily. These areas typically have a high reliance on locally based primary health care (PHC) facilities with small permanent staff numbers (often restricted to physicians and nurses) and ancillary services (allied health, dental health, mental health) provided by visiting or locumpractitioners. Service delivery also features frequent demands for users to travel within and out of the area for even relatively minor treatments (including diagnostic imaging and bloodwork) (3, 4). The incidence rates of COVID-19 can be found in Figure 1 below (5). USA has the highest incidence of COVID-19 (measured in daily cases per 1 million people). Sweden follows, with Canada and Australia third and fourth, respectively. Figure 1 clearly displays the spikes in daily case loads associated with waves of COVID-19 infection. The ethnography completed in this study occurred during the summer of 2020, which coincides with the second wave of COVID-19 infection world-wide. Of particular note is the recent change in case load within Australia and Sweden. Differing COVID-19 management strategies led to Australia having relatively low case numbers for the better part of 2 years. Sweden meanwhile adopted a herd immunity tactic, which led to case numbers per 1M people rivaling that of the USA. In the fourth wave of the fall of 2021 however, Sweden case numbers dipped below Australia's for the first time since the beginning of the pandemic.

The ability for local health and care services to act somewhat autonomously in responding to health risk events like the pandemic should be seen as a critical part of socially responsible and community-based care paradigms (6–8). These paradigms emphasize the need for services to understand the communities in which they operate and to tailor what they do to the needs of those communities (1). Given the diversity of rural communities (9), this means that services in even relatively proximate communities could and should operate differently to one another. There is of course a tension between allowing sufficient local autonomy to develop community responsive service models and maintaining regional, provincial, or national standards (10), and part of the value of this research is contributing to understanding and potentially resolving that tension.

While many health risk events are largely unexpected and require rapid response, the COVID-19 pandemic has presented some specific challenges (11). It has been a protracted event, with health service delivery substantially affected for over a year at the time of writing. It has been a geographically widespread event, with global impact. Further, it has been an event which has directly impacted health service delivery, causing changes to how and where and what services are delivered (12). Our study addresses the gap in knowledge with regards to rural health system innovation in the face of an unprecedented stressor such as COVID-19. This ethnography sought to document—in real time—the responses to COVID-19 and codify them for future reference and dissemination as policy reforms for rural health systems and services. Creating a body of literature for rural practitioners, especially with regards to what worked for other communities in diverse contexts both geographically and system-wise, could strengthen rural health systems as the COVID-19 pandemic marches on.

## Methods

The research was conducted from a service user perspective, involving ethnographic and observation-based methods employed by members of the research team who were residents of or visiting communities at the time. While researchers were asked to position themselves as if they were a service user, it is important to note that all members of the team were in some way associated with the health and care sector (although not necessarily in the communities where they conducted research)—as practitioners, students or researchers. The team employed both a recursive (13) and discursive research approach, with regular sharing of ideas and insights between team members in the same country and between countries guiding what was done next and the development of frameworks for data capture and analysis.

The purpose of those quick village vignettes was to provide a glance at what health and care services are available and what are restrictions had been implemented due to the COVID-19 pandemic. We selected several villages from regions in Australia, Canada, Sweden and the USA for comparison and inclusion in the dataset, each following a similar methodology (14). The inclusion criteria of these sites was largely open-ended, with consensus from the group part of the process in determining if a site was appropriately rural. Proximity to these sites by our group also figured into their selection, as the logistics of conducting an ethnography in the midst of the second wave necessitated streamlining various factors such as the ability to assess community response and familiarity with systems. The resultant vignettes from selected villages offer a contemporary snapshot of the efforts service providers made within the context of broader shifts in health and care delivery.

The overall methodology for the village vignettes was as follows: we briefly described selected case sites in terms of their eHealth development and institutional arrangements for local health and care systems *prior* to the pandemic. Next, we briefly described the jurisdictional (national and provincial) eHealth responses to the pandemic which are particularly relevant to small rural health and care systems in the context of the macro factors (demography, economy, accessibility) already influencing design and redesign of these systems. Following, we provided examples of how health and care systems have been affected by the pandemic in specific communities, looking particularly for “extreme” cases which show either innovative engagement in new ways of working at the local level, or substantial challenges faced. Lastly, we use the rural eHealth literature, the experiences of the case sites, and our own experience as health and care professionals, researchers, and educators during this period to identify issues arising from the rapid expansion process which local systems need to consider when planning eHealth beyond the pandemic.

Building from the above, we developed a methodology of what we did for each village, with the recognition that there will necessarily be some variation given local contexts. When conducting our investigations, we were guided by an impact domain framework which sought to holistically evaluate the COVID-19 response. This framework includes patient risk (continuity of care, inclusive care, accessibility), service design and innovation (empowering local service managers and communities, service integration), workforce (recruitment, retention, education and training), the technology itself (compatibility, usability), and stakeholder engagement (government agencies, private health and care providers, universities). The framework identifies how the rapid expansion of eHealth services might provide benefits and mitigate negative impacts in these domains, offering suggestions as to how small rural systems might respond to the challenges and use this opportunity to improve the provision of health and care services in what might be considered marginal environments. This framework prescribed the themes which guided our ethnography at these various sites (15, 16).

For each village, we followed a similar set of guidelines where we examine health and care services across a range of factors, developing a quick “picture” of what might be available to residents. This methodology is in essence a remote access version of a village observation protocol, where we take a glance at villages from the outside and look for indicators of activities such as migration, employment, or social connection. Here we are looking at the range of health care services in a village, what was present before the current pandemic, and how these may have shifted in the recent months. With our impact domains of patient risk, service design and innovation, workforce, and stakeholder engagement in mind, each village facet was examined through the following methodology:

**Table:** 

**Service**	**Elements**	**Sources**
Context	Distance to larger center Distance to hospital Number of physicians Population & pop. change Other relevant context	Websites & Google Maps Statistical Agency Social media
Physician	Availability Booking (phone, online)	Websites/Social media Clinic phone messages
Hospital	Emergency Services Walk-In Clinics? Testing & blood clinics Routine Clinics	Websites Phone messages
Public Health	Presence of Public Health Unit Information on COVID-19 Updates & Relevance	Social media Websites
Mental Health	Walk-In/Telephone Services Counselors & Psychologists	Social media Websites Listing on other sites
Municipal Services	Services available/limited Face-to-face, telephone Online systems Updated information	Website Phone message Social media
Social/Community Services	Home-care services	Websites
Other Care Services	Long-term care facilities Physiotherapy Dental, orthodontics, denture	Websites Phone messages Social media

### Basis for Comparison

While our research was conducted in broadly similar geographic contexts in the four countries, the selection of those countries and the specific case sites within them was largely opportunistic, being where members of the research team were located or had regular access. Given the guiding methodology was under an ethnographic paradigm, it bares examining the bases for comparison encompassing both similarities and differences.

At a political level, Sweden's (at least the parts of Sweden where this research was conducted) health care system is almost entirely publicly funded and administered (17). There are private practitioners (mostly locum service providers) but they are contracted to the public system. Australia and Canada have similar public-private service models involving fee for service reimbursements from public and private health insurance providers (18, 19), and a mix of public and private services. The USA largely relies on private provision of health care, with minimal public insurance and government-operated services (20).

All countries have highly regulated health sectors, with national systems for approving pharmaceuticals and treatment methods, and strong medico-legal systems. However, all countries also have complex health system structures particularly in rural areas (21). The complexity arises from the interactions between public and private sector actors, and even more from the division of responsibilities between different levels of government (national, provincial, and local). This is perhaps most extreme in Sweden where local government has direct responsibility for the provision of aged care, home-based care, health services in (junior) schools and other community-based services.

Local government does not have such responsibilities in Canada (22) or Australia, but there is a division between provincial and national government responsibilities with provinces managing (among other things) hospitals and emergency services, and national government managing medical workforces, health insurance and regulatory frameworks. Provincial and even local governments in the USA have legislative power to intervene in health service administration and delivery and do so in different ways depending on political orientations. The configuration of health care services can differ dramatically even in relatively proximate locations in the USA. In all countries, there are regular debates between levels of government about health care funding and responsibilities and concerns about lack of coordination between levels of government (and government and private providers) that lead to duplication of services and substantial service gaps (22, 23).

In Australia and Canada, towns at the larger end of our size spectrum are likely to have both a general practice/ family practice clinic and a hospital with limited functionality [aged care, rehabilitation, triage (24)]. Swedish rural sites provide care through a “cottage hospital” (*sjukstuga—*plural *sjukstugor)*. Occasionally there may be separate facilities for dental health (or physiotherapy or mental health), but usually non-GP services operate out of the hospital and are delivered part-time. Smaller towns may have a general practice clinic operating part-time. There are privately operated pharmacies in larger towns. In small rural Australia and Canada, accessing health care almost always involves visiting the hospital or the clinic. Rural hospitals are always at risk of closure and reduction of services (25).

The United States has a model of care different from Sweden, Australia, and Canada (26). This private model of care means that most services which are subsidized by taxpayers in our other three countries require out of pocket expense if an individual in the United States does not have private health insurance. In the north-eastern United States, where our vignettes were based, there is a large telehealth service which connects rural physicians with specialists at a larger urban hospital. Having very good existing technological infrastructure aided rural Americans in New England in their transition to online models of care at the onset of the COVID-19 pandemic.

## Results

Access to service is assessed through reporting of how these rural villages and towns handled service provision during the onset of COVID-19. Additionally, access was assessed by evaluating information such as resource availability and mechanism of delivery. They were recorded by researchers who lived and worked in these communities.

### Canada

The Canadian rural response to COVID-19 was measured in two separate contexts from both a quantitative perspective (to ascertain demographics) and a qualitative perspective (to analyze actual practices implemented to expanding and changing care). The province of Ontario and the province of Nova Scotia were chosen as suitable candidates to draw sites from, mostly due to the proximity of our research group to these provinces, and the access to existing contacts and circles already established in previous research projects.

It is worth noting that as of the time of writing this paper, Nova Scotia and Ontario have had much different experiences in managing the COVID-19 pandemic. Nova Scotia has seen great success'bubbling' with neighbor provinces in the Canadian Maritimes. Besides the odd outbreak over the summer of 2020, trends of COVID-19 spread in Nova Scotia have been extremely small. Ontario however began it's third wave of COVID-19 spread in early March 2021 and entered a 28-day provincial wide lockdown on April 3rd 2021 to flatten the curve of COVID-19 transmission. Acknowledging this is important, because much of the services in Nova Scotia could be provided mirroring their service prior to COVID-19 disruption.

With regards to service provision, both provinces responded to the COVID-19 pandemic with changes to service protocols, but Ontario was far more drastic and longer-lasting in their approaches. For example, In Nova Scotia cancer care was continued, while in Ontario, some treatments and surgeries were delayed. Many elective surgeries were delayed in Nova Scotia at the outset of the pandemic, but as they successfully flattened the curve treatments which had been postponed were rescheduled promptly. Ontario had to postpone much of their elective surgeries, and as Ontario enters a third wave, many services which had been postponed over a year prior still have not seen a return to their implementation prior to the pandemic.

Further, Nova Scotia has one web domain with all health centers throughout the province included, with updated information during COVID-19. While Nova Scotia had one web domain for most major hospitals and clinics, this domain was *not* linked to most family physician offices. Family doctor offices provided links to governmental resources for patients, but rarely did they update their own websites. Ontario has individual websites per health centre which are updated at the discretion of that health centre, meaning some haven't been updated in years. This has made getting service in rural communities difficult, and there is no clear avenue to see who the appropriate person is to approach about getting information regarding up-to-date information for health centers. Centralizing health center informational streams on one domain expediates the process of informational exchange, and allowed for current displays of protocols, progress, and changes to service provision. The response from larger urban health centers were generally the same in both Ontario and Nova Scotia. No visitors, redirection of patients to other services. Non-urgent medical tests were pushed. These include screenings and medical imaging. Many centres stopped taking drop-ins but were still seeing appointments.

One very encouraging practice which came out of Ontario was the County Virtual Triage Assessment Center (CVTAC) was developed in an effort to redirect patients using the emergency department/hospital for things that can be provided by a family practitioner, such as prescription refills. The goal of the implementation of CVTAC was to strengthen access to primary care, as per the county's webpage. It's goal was to reduce the demand on the emergency department, and its prolonged implementation can only be beneficial in combatting emergency department overcrowding into the future post COVID-19 pandemic. Without the CVTAC, the primary care which was available in rural Ontario was difficult to access before the pandemic and became near impossible during it. Unfortunately, the funding which support CVTAC is tied to COVID-19, and will likely disappear once vaccinations begin to ramp up. Technology and innovative strategies like CVTAC need clear funding sources moving forward, as creating an inherent clause in their implementation to roll them back post COVID-19 is damaging to the overall rural health system they were introduced into.

Group services, much as in Sweden and Australia, saw a pause in most communities, but there were some progressive community groups which relied on volunteers to perform group activities which existed before the pandemic, and create novel activities during the pandemic to combat social isolation. These community volunteer groups were usually (but not always) faith based and did not have external funding. There was minimal guidance or recruitment for official group activities run by either health authorities or public health offices in rural Ontario and Nova Scotia.

While Nova Scotia has a smaller population, the concept of having one health information source (one website) ensures that the entire province is on the same page, in terms of response to COVID-19. It also ensures that there is up to date and clear communication from all health centres, as they all fall on the same website. This also lessens the confusion as to what is a reliable source. While health centers benefitted from uniform messaging across sites, individual physician offices or webpages did not update their information regularly. Most sites were out of date, and those which were current did not provide any specific information for their context, and instead referred patients to the larger Nova Scotia web page for health centers.

In Ontario each health centre has their own individual website (much like each individual family physician office in rural Nova Scotia). This was a problem in Ontario as health center websites are more prominent and were consulted more frequently for information. Many of the websites being looked at in the vignettes, were dated and unreliable, with no current information on COVID-19. Many had more reliable and up to date social media accounts (Facebook, Instagram and Twitter). There were also instances of social media accounts for the health centres that were run by members of the public, not associated professionally with the health centre. While most of these accounts were run in good faith, there is of course the possibility that these accounts could post information to craft a narrative of disinformation, which existed during the pandemic if not monitored by an official source. This makes for a more difficult search to find information, leading people to call or go to centres to find out more information. Or avoid centres even if they are sick, due to the unknown measure put in place to protect those without symptoms of COVID-19.

### USA

American COVID-19 response was measured using the same metrics as the Canadian context. Vignettes were chosen from north-eastern United States, in the Vermont and New Hampshire areas. Again, like the Canadian, Australian, and Swedish contexts, these communities were chosen because of their proximity to our research cluster. These communities are likely not a good representation of the average community in the United States, as their median income is much higher than other states. Their affluence may be part of the reason the infrastructure and services available to them are better, relevant to their Swedish, Canadian, and Australian counterparts. The private nature of the American healthcare system also means these households can dedicate more of their income to their health and will probably be able to access services which many rural communities cannot.

All the vignette sites had access to family physicians, while only one provided the access through a regional hospital. Most sites increased their service provision during COVID-19 through a hybrid approach of telehealth and online services. There was already an existing service which connected rural physicians (and by extension, their patients) to a dumber of specialists at a larger level 1 trauma center in New England. This likely smoothened the process of change of service protocols, as much of the precedent and comfort of working through eHealth existed in the area.

Communication at the American vignette sites was good, with most of the vignettes having up to date websites regarding COVID-19 protocols. Additionally, most had a way to notify the public when protocol changes, with social media accounts run in conjunction with clinics and health centers in the area. When compared to Ontario and Nova Scotia, the New England sites were not all on one domain like the Nova Scotian centers, but they weren't quite as diverse as the Ontario sites. All sites seemed to be run with some leadership and direction, but this could not be confirmed from the information provided. Commonalities such as phrasing and links to other resources however point to some co-ordination in messaging and keeping the information current was common to all vignettes chosen. Outside of that, much of the broad responses to the pandemic were the same in the United States as they were in Canada, Australia, and Sweden—pushed elective surgeries, physical distancing, and limited visitation.

Interestingly, much of the response in the New England hospitals seemed to be on a consultation basis, with numerous clinics and hospitals stepping up their public health footprint during the COVID-19 pandemic. They offered information broadly on how to avoid the virus, but one site also offered information which would be unique to that site's context—namely, how to reopen small business again safely and successfully, following government mandates and health outlines. This tailoring to community concerns is a positive outcome seen in the other countries analyzed, where at their best rural health centers become resources for things other than strictly health guidelines. Becoming trusted centers of information for things such as small business protocols was a positive reinforcement of the beneficial standing most of these centers have in their communities.

Another positive of the United States rural COVID-19 response was the focus on mental health. Like other health services, much of the mental health programming was transitioned into a telehealth or eHealth medium at the onset of the COVID-19 pandemic. There was however a conscious effort, as evidenced by resources online and through social media content, in reaching out to patients regarding their mental health and ensuring that they knew their options. In comparison with the rural Canadian sites, the focus on mental health in the United States was coordinated across organizations and health centers and prioritized by health authorities.

### Australia

In Australia, the most striking phenomena was the contrast in responses from primary care facilities that were quite proximate to one another, and in one case even had clinics in the same town. In one case, all that was offered was a handwritten sign on the clinic door saying to call for an appointment or attend during reduced hours. No website or social media presence, no further information. Once you called the number or presented in-person, you got the treatment you were looking for—renewing a prescription or a similar service—but it did seem like the clinic was somewhat divorced from the community. In contrast, we saw other clinics who seemed less narrowly concerned about their own business (making sure they had access to their patients) and more concerned about their role in the community. They became the main sources of credible local information about COVID-19 and about how you could navigate the health and care system while the pandemic restrictions were in place. Like in the north-eastern united states, this was a positive outcome of COVID-19 response. Improving their visibility in the community meant having clear signs at the clinic, on community noticeboards, on their own websites and social media, and on other websites and social media that the community were likely to use.

We also saw some of these clinics expand their scope of practice, or at least engage in different activities or do them in different ways to what they had done previously. The “public health consultant” role was a clear one—in the past this may have been a passive role involving brochures and posters at the clinic, but now was a service you could access by calling the clinic and getting advice about community-based services and their operations during COVID-19. There were also cases of local services delivering public health messages in novel ways (through musical performance, for example) which increased the reach of information. This was particularly important for mental health related issues.

There were other forced changes that had the potential to be handled better. One was the interruption of group-based treatments. Groups obviously couldn't meet face to face, but the only alternatives we saw were instructions to call a certain number for a one-to-one consultation if you felt you needed it. Similarly, patient transport services were interrupted, and people who did have to travel for advanced care either went without that care or had to find an alternative with not much help to do that. We saw something similar with respite care suddenly being inaccessible and obviously creating problems for patients and their caretakers.

Aspects of navigation through the system did seem to be well-addressed. A particular example is the apparent streamlining of processes between the clinic and the pharmacy. In the past, the patient needed to take the prescription physically from the clinic to the pharmacy, and then the pharmacist might have to check with the physician and so on. But at least in a couple of cases we saw the clinic communicate directly with the pharmacist, so everything was ready for the patient when you arrived at the pharmacy. Again, this worked well for people who were well known by the clinic and the pharmacist but may not have been so functional for more marginalized members of the community and was not standard practice across all clinics. We also saw an increase in whole-of-family services, the most notable being scheduling influenza vaccines for the whole family at once rather than one person at a time. Often, this was done on a “drive-through” basis with clinic car parks and public spaces becoming temporary consulting rooms.

In general, we saw that clinics could and did do a lot to ensure that their own services to their own users were not just maintained, but even enhanced by things like teleconsultations and streamlined referral processes. We saw that clinics could and did assume roles as community leaders in the provision of local and general information about the pandemic and how to access care during the pandemic. We saw more use of telehealth rather than eHealth, in the sense that virtual consultations were by telephone and audio only rather than by videoconference.

In summary, the evidence we had was that primary care services which were well-connected with the community and who saw their responsibilities as extending beyond providing their normal fee-for-service activities were able to exercise leadership and implement new ways of doing things. This not only minimized disruption but enhanced quality of care and efficiency of care provision within a short time frame. Further research is needed to understand how vulnerable or marginalized populations were supported, and to see how local services managed their relationships with provincial health departments, distant specialists, and other external actors. Our impression from the limited exposure we had to these latter was that they were simply waiting for things to “return to normal” rather than investing too much in adapting their services during the pandemic.

### Sweden

Parts of rural Sweden is known for its history of health service innovation, particularly in the use of eHealth. There's documentation of eHealth developments over at least the last 30 years (27), and in recent times the region has received academic attention for novel methods of delivery primary care services in communities without health services (28) and for local engagement in medical education (29). Some of this innovation has come “top down” from the provincial health department, but quite a lot of it has come “bottom up” from local health services, particularly in the municipality of Storuman, where a physician established a “Centre for Rural Medicine” some 10 years ago (30). In some ways, then, services in this region were reasonably well set up to deal with the challenges presented by the pandemic. Teleconsulting was already common, including teleconsulting for emergency and primary care. Most health services already had pretty high-quality video-conferencing facilities. Electronic prescriptions, electronic referrals (and teleconsulting with distant specialists), digital platforms for booking appointments, remote imaging (ultrasounds, dermatology etc) and other “doctor at a distance” techniques were widely used and quite well-understood by service providers and users.

Health and care services in this region have been used to operating in crisis mode and this, along with the relatively late arrival of the COVID-19 virus in the rural communities here (very few cases until October 2020) perhaps contributed to a complacency among providers and users. Adapting to recommendations to limit physical contact was quite easy since the sorts of techniques to facilitate that were already widely used. Nevertheless, we did see some of these practices become more entrenched in locations which had not used them so much previously, and more support came from provincial and national health authorities for embedding these practices in primary care services. One of our research team noted that stakeholders were somewhat surprised at how quickly health authorities were able to change procurement procedures and other administrative aspects that had contributed to a reputation of a slow-moving public health and care system. Those central innovations then allowed some local services to enter partnerships with technology providers and trial models of service delivery (including virtual clinics with the physicians located in other parts of Europe) that might have taken much longer to put in place prior to the pandemic.

Generally, though, what we saw at the local level was not so much innovation as extension of practices that were already being established. The process of moving from heavy reliance on expensive locums to provide in-person services to increasing use of digitally mediated services as a COVID-19 response to local workforce shortages had already begun but was accelerated by the pandemic. While this meant that local services could continue to mediate “good and close care” (in terms of limiting the need for patients to travel) as required by the Swedish national policy, it also meant that the physical distance between communities and care providers increased.

There were some signs of breaking down of barriers between municipal and provincial services. In one case at least, provincial staff were redeployed to municipal-run aged care facilities rather than the municipality being forced to acquire increased debt to bring in “emergency” staff from outside the region. This did mean that other parts of the system were left unstaffed, or staffed by unqualified personnel. This was particularly difficult in municipalities which had previously invested heavily in supporting in-home aged care through frequent home visits by district nurses and others. Nevertheless, it demonstrated that cooperation between levels of government was possible, and that such cooperation could be initiated locally.

## Discussion

### The HOW Framework

Local autonomy in rural health can be compromised by structural factors such as workforce turnover, limited funding, tensions between levels of government, medico-legal concerns, lack of access to information and knowledge, latent inertia and risk aversion. In highly regulated systems it may be difficult for services to act locally to implement initiatives that have not been centrally mandated. This may particularly be the case in rural areas where services are often fragile because of difficulties in recruiting and retaining professional staff, insecure funding and a tendence by regulators to more closely monitor policy compliance among services they rarely physically visit. Discouraging local action may increase in times of crisis as regulatory agencies implement centrally managed crisis management plans.

There is evidence, however, of locally driven and novel initiatives in rural health in each of the four countries (11, 31). The CVTAC in Canada, the adaption of health promotion responsibility and material in all four countries, and the technological adjustments to prescriptions and services by pharmacies and rural medical centres are three examples from our field research which point to an ability of rural communities to innovate in the face of system stress. A number of telehealth initiatives, and mechanisms for cooperation between local and provincial government have all emerged from within services based in rural areas. In all four countries, changes in rural health care systems usually result from pilot studies or limited trials which expose local services to “doing things differently” from neighboring services and building capacity as leaders and participants in innovation and reform, but with the tension of change being so high, innovation and reform become the norm of operating in rural communities.

There is a growing literature on innovation in rural health which has a focus on locally driven initiatives (32–34). The literature mostly focuses on the innovations themselves rather than the mechanisms which enable innovation (or “autonomous action” in terms of choosing to do things differently). That literature did, however, provide an initial program theory of how local health services could direct their own responses to the pandemic. Clearly there needs to be a *policy environment* which allows or even encourages local actors to make decisions about relevant aspects of service design and delivery [whatever those aspects might be (35)]. This could be seen in all four regions we conducted our ethnography, as the policy called for restrictions and temperament in services provided. Health centers responded by implementing innovating services to continue group care in Sweden and Australia, and setting up uniform points of contact and information in USA and Canada.

There have been endless calls for moves away from “one size fits all” service models, with assertions that policy which focuses on outcomes (accessibility and health outcomes) is likely to be more effective than policy which focuses on inputs. There also needs to be *local leadership* and champions, as seen in the implementation of the CVTAC in Canada, where imagination and creativity allowed local actors to recognize good ideas when they see them, and coordinated their implementation and ongoing operation.

This leadership is central to what is known as innovation capacity. The innovation literature (which rural health academics have only begun to recognize as applicable in their contexts) also talks about *absorptive capacity*, which is about building knowledge of what is possible and evaluating options in terms of their fit to the needs of the community. To that end, this ethnography serves as a primary investigation into the viability of performing a realist review examining what works where and for whom for innovative strategies in rural health services. Sites in Canada, Australia, and Sweden had diverse responses all of which conformed to the universal healthcare paradigm, and could be referenced in future with regards to rural health policy reforms.

Such knowledge should equally recognize why particular initiatives that appear successful elsewhere might not work in a particular location. There needs to be *collective mechanisms*, through which local actors engage with their communities and manage partnerships with external actors (including policy makers and “downstream” service providers). More recent literature has talked about an ability to implement change in such a way that perverse outcomes can be quickly identified and responded to and a process of continuous improvement can be undertaken which also recognizes unforeseen opportunities. This has been referred to as an *antifragile design* approach (36, 37).

### The WHAT Framework

The pandemic brought with it changes in models of service delivery mandated by government health departments (38, 39). Largely these were around minimizing physical contact between service providers and users, so there has been a lot of attention paid to eHealth applications, automating paperwork processes (such as electronic prescriptions in places which were not already using those), and reducing drop-in type services. Even here, though, there is scope for locally diverse action to ensure that implementing pandemic-inspired regulations did not unnecessarily reduce access to care and quality of care. Similarly, public health guidelines (such as minimum distances between people in various settings, conditions under which one might seek a COVID-19 test, maximum number of people for group-based activities) have needed to be interpreted at a local level.

A question for this project was not just “what sort of responses might local services implement?,” but “what sort of responses would be visible to communities/service users?”. The innovation literature in rural health (40, 41), such as it is, identifies three main types of local action –

1. Adopting (and very occasionally inventing) eHealth technologies;
2. Changing service structures
  - Having different services or types of professionals change how they work together
  - Changing how physical infrastructure is used
  - Establishing a configuration of services targeted at specific populations or health conditions
3. Changing funding models or how funds are used locally.

In “normal times,” these changes tend to occur over long periods of time, favoring “prudence” over “speed” (42). With the pandemic, however, rapid change was required, meaning that service managers had to quickly draw on their absorptive capacity. The speed with which new ways of doing things were implemented therefore reflects this aspect of the HOW framework and sits above the WHAT framework as “*evidence of preparedness*.” In our research we had cases where health services (such as group counseling services) closed completely for a period without an alternative offer, indicating low levels of preparedness, and other cases where information about new processes and procedures was provided to the public almost as soon as new regulations were announced.

This provision of information is the cornerstone of WHAT local actors could do effectively. Information provision responses were of two types. The first was to inform community members about changes in how services were provided and accessed. One barrier to accessibility of rural health services is a division in the community between those who have the tacit knowledge about how the system works and how to access it, and those who do not. At the start of the pandemic, this division temporarily disappeared. We could then observe how local services distributed *guiding information*.

The second information impacts were local services taking on new or expanded *public health information* provider roles. Typically, public health information exists as standard (i.e., sourced externally in a standard format) brochures or posters inside health and care facilities or on community noticeboards. Rural communities are also often engaged in externally funded public health campaigns which may be implemented through local services, but typically involve outsiders visiting “the community” (school, aged care facility, community group) and doing presentations or workshops. These sorts of “pre-packaged” approaches were not able to keep up with community need for quickly provided information about the pandemic and its local impacts.

Guiding information was often necessary because of the *physical and procedural changes* that were made to service delivery. Within a care facility, this might have entailed new methods for making bookings (from a distance rather than in person), new ways of managing appointments (arrival and departure procedures, uses of waiting rooms), and changing the physical layout of the facility. There could also be changes in how care activities were distributed among the set of facilities that exist in a community (including non-care specific facilities like schools, meeting halls and so on).

The provision of timely, locally relevant, and broadly accessible information could of course be facilitated by changing how *digital communication technologies* were used. There was also a sense in which the pandemic “released the shackles” on using digital technologies in the actual process of care provision. Long persistent barriers to employing eHealth such as provider and user reluctance, regulatory and financial structures, concerns about quality of video and audio links and so on were swept aside as if by magic and non-contact care models were not just encouraged but mandated situations. Locally, service providers needed to quickly develop their own eHealth skills and help users to do the same. Local services could also choose to employ eHealth beyond the minimum if they saw opportunities to go beyond what was mandated.

Physical and procedural changes in service provision models and changing use of eHealth impacted *coordination between service providers* (and other stakeholders) within the community and external to the community. From a user perspective we could observe how “journeys” which involve a number of different providers were managed and the role of local actors in facilitating those journeys.

## Conclusion

The lessons we can draw from the vignettes of rural health and care systems presented in this article follow along two lines: theoretical and operational. Theoretically, the COVID-19 pandemic has resulted in extreme levels of stress on local health and care systems and our evidence has shown examples of where these have flourished and provided new models of care or new services for rural communities. Rural organizations are well-conditioned to uncertainty given often limited and temporary funding, high turnover of the professional workforce, and shifting priorities of regional and state and/or provincial governments. As such, they have developed a high absorptive capacity given the need to adapt to frequent change.

Operationally, three key features have come forth as being paramount to successful innovation and response in rural communities and care systems, captured in our WHAT and HOW frameworks. First, there needs to be a high degree of collaboration and connection. This collaboration is not only internal to the communities themselves, but also with government at higher levels, private business, and social enterprises. Much of these connections already exist in the small places we studied, but our successful examples all included collaboration from numerous stakeholders. Second, there needs to be a high level of familiarity and knowledge of *local* environments. The axiom that all rural communities are unique appears to hold true, where knowledge of how services are used, who provides them, and who uses what services is essential to program success and adaptation. Third, there needs to be creativity in how limited resources can be managed and adapted, including using new technologies. The most successful examples we profiled responded to a resource shortage with new technologies and an adaptation to the local community context.

This article has shown the potential for innovation in rural communities and in rural health and care systems. Rural health and care systems can be loci of adaptation and innovation given the appropriate mix of local autonomy, strong service-community connections, high absorptive capacity, and evidence of organizational antifragility.

## Data Availability Statement

The raw data supporting the conclusions of this article will be made available by the authors, without undue reservation.

## Author Contributions

SP, DC, and PP wrote and edited the manuscript. SP, DC, PP, A-KH, HH, ML, HS, MO, JB, MGG, MS, and JG performed research and contributed their findings to the manuscript. All authors contributed to the article and approved the submitted version.

## Funding

Contributions from DC, A-KH, and MGG were funded by the Swedish Research Council for Health, Working Life and Welfare (2017-00183).

## Conflict of Interest

The authors declare that the research was conducted in the absence of any commercial or financial relationships that could be construed as a potential conflict of interest.

## Publisher's Note

All claims expressed in this article are solely those of the authors and do not necessarily represent those of their affiliated organizations, or those of the publisher, the editors and the reviewers. Any product that may be evaluated in this article, or claim that may be made by its manufacturer, is not guaranteed or endorsed by the publisher.
